# Do the correlates of screen time and sedentary time differ in preschool children?

**DOI:** 10.1186/s12889-017-4195-x

**Published:** 2017-03-29

**Authors:** Katherine L Downing, Trina Hinkley, Jo Salmon, Jill A Hnatiuk, Kylie D Hesketh

**Affiliations:** 1 Institute for Physical Activity and Nutrition (IPAN), School of Exercise and Nutrition Sciences, Deakin University, Geelong, Australia; 2 School of Science and Health, Western Sydney University, Penrith, NSW 2751 Australia

**Keywords:** Sedentary behaviour, Sedentary time, Screen time, Preschool children, Paediatric, Accelerometry, Television viewing

## Abstract

**Background:**

Preschool children can spend up to 12 h a day in sedentary time and few meet current recommendations for screen time. Little is known about ecological correlates that could be targeted to decrease specific versus total sedentary behaviour. This study examined whether the correlates of screen time and sedentary time differ in preschool boys and girls.

**Methods:**

Parents participating in the HAPPY Study in 2008/09 in Melbourne, Australia reported their child’s usual screen time and potential individual, social and physical environment correlates. Children wore ActiGraph GT1M accelerometers for eight days to objectively assess sedentary time (<100 counts.min^−1^). Multivariable linear regression analyses were performed, stratified by sex and controlling for child age, preschool/childcare attendance and clustering by centre of recruitment. Correlates significantly associated with screen time or sedentary time in individual models (*p* < 0.05) were included in final combined models.

**Results:**

Children were sedentary for 301.1 (SD 34.1) minutes/day and spent 108.5 (SD 69.6) minutes/day in screen time. There were no sex differences in screen or sedentary time. In the final models, sleep duration was inversely associated with girls’ sedentary time and boys’ screen time. The only other consistent correlates for boys and girls were parental self-efficacy to limit screen time and screen time rules, which were inversely associated with screen time for both sexes. Parents reporting that they get bored watching their child play was inversely associated and maternal television viewing was positively associated with boys’ screen time. Paternal age was positively associated with boys’ sedentary time. Maternal ethnicity was inversely associated and paternal education, child preferences for sedentary behaviour, and parental concerns about child’s physical activity and sedentary behaviour were positively associated with girls’ screen time.

**Conclusions:**

The modifiable correlates of total sedentary and screen time identified in this study could be targeted in interventions to reduce these behaviours. With correlates differing for screen and sedentary time, and between boys and girls, interventions may also benefit from including behaviour- and sex-specific strategies.

**Electronic supplementary material:**

The online version of this article (doi:10.1186/s12889-017-4195-x) contains supplementary material, which is available to authorized users.

## Background

Sedentary behaviour, defined as any seated, waking behaviours requiring ≤1.5 Metabolic Equivalent of Tasks (METs) to perform [1], can include watching television, playing electronic games and reading. Sedentary behaviour research to date has generally focused on screen time (i.e., the sum of time spent viewing television, playing electronic games, and using a computer or other electronic devices) and, to a lesser extent, sedentary time (objectively assessed, e.g., by accelerometry). Sedentary behaviours have their genesis in early childhood (birth through 5 years of age) [2]. While there is currently no evidence for negative health consequences of sedentary time in early childhood [3], excessive screen time has been associated with poorer cognitive development and well-being, and increased risk of overweight and obesity [4–6].

A recent systematic review found that preschool children (roughly 3 to 5 years) spend up to 12 h per day in total (objectively measured) sedentary time [7]. Additionally, research suggests that preschool children spend an average of two hours per day engaging in screen time [8–11], with approximately one quarter meeting current recommendations of less than one hour of screen time per day [8, 9, 12]. Given the low level of compliance with screen time recommendations and high levels of sedentary time, it is important to identify the factors that are associated with specific and sedentary time in young children in order to inform the development of appropriate intervention strategies.

A systematic review of correlates of sedentary behaviour in preschool children found that studies investigating potential correlates of sedentary behaviour have largely examined television viewing only, with very few investigating correlates of overall screen time or sedentary time [13]. That review also found few consistent correlates of screen and sedentary time. A more recent review examining correlates of energy balance-related behaviours in preschool children found that parental body mass index (BMI), family size, higher energy intake, consumption of high energy drinks, consumption of savoury snacks, parental television viewing time, the presence of a television in the bedroom, having a cable subscription and the day of the week (weekdays) were positively associated with screen time, while fruit consumption and living in an urban region were inversely associated with screen time, with no differences in correlates between sexes reported [14]. However, that review focused exclusively on screen time, with correlates of sedentary time not reported. Studies investigating sedentary time in preschool children have reported that girls are significantly more sedentary than boys [15, 16]. Further, television/video games and physical activity equipment in the home were also shown to be positively associated with sedentary time for boys, while child BMI z-scores and parent-reported athletic coordination were significantly associated with sedentary time for girls [15].

No studies have been identified that examine the correlates of screen time and sedentary time in preschool children in the same sample; however, recent research of this nature in 9- to 11-year-old children has shown that the correlates of these behaviours differ [17, 18]. This suggests that there is a need to investigate screen time and sedentary time as separate behaviours, potentially requiring different strategies to decrease time in those behaviours. Moreover, research has shown that correlates of sedentary behaviours differ between the sexes in preschool [15, 19] and school-aged [17, 18] children, suggesting that correlates should be investigated separately for boys and girls. The aim of this study was to investigate whether the correlates of screen time and sedentary time differ in 3- to 5-year-old boys and girls.

## Methods

### Recruitment and participants

This study used baseline data (from 2008/09) drawn from the Healthy Active Preschool and Primary Years (HAPPY) Study when children were 3 to 5 years old. HAPPY is a cohort study, conducted in Melbourne, Australia, that investigates multi-domain correlates of physical activity and sedentary behaviour. Recruitment and data collection for this study have previously been described [20]. Briefly, two local government areas (LGAs) within each of the lowest, middle and highest socioeconomic quintiles in metropolitan Melbourne were randomly selected (six in total). Within each of those LGAs, once permission was granted, 124 preschools and 146 childcare centres were randomly selected and invited to participate. All parents (*n* = 9794) of children aged 3 to 5 years at consenting preschools and childcare centres were then invited to participate in the study. Data were collected from 1002 children and their parents (11% response rate). The final sample included 937 children (*n* = 504 boys) with valid screen time data and 724 children (*n* = 397 boys) with valid accelerometry data. The Deakin University Human Research Ethics Committee and the Department of Education and Early Childhood Development approved the study.

### Measures and data management

#### Outcome variables

Children were fitted with ActiGraph GT1M uniaxial accelerometers (Pensacola, FL, USA) on an elastic belt at the right iliac crest and instructed to wear them during waking hours for 8 consecutive days to objectively assess sedentary time. ActiGraph accelerometers have established validity and reliability in preschool-aged children [21]. Data were collected in 15-s epochs [22, 23] to account for the sporadic nature of young children’s physical activity. Non-wear time was determined as ≥10 min of consecutive zero counts [24]. Sedentary time was classified using Evenson et al. [25] cut points of ≤25 counts per 15-s epoch. To be included in the analyses, children were required to have data recorded for at least 6 h per day on at least 4 days (including at least 1 weekend day) [24]. To account for variations in children’s accelerometer wear time, sedentary time was standardized using the residuals obtained when regressing sedentary time on wear time [26].

During the week that children wore the accelerometer, parents completed surveys reporting their child’s usual television/video/DVD time, computer use, and sedentary electronic game use (in hours and minutes) on weekdays (i.e., total time from Monday to Friday) and on weekend days (i.e., total time on Saturday and Sunday). Responses were converted to minutes, then weekday and weekend responses were summed and divided by seven to give average daily minutes of screen time.

#### Explanatory variables

Explanatory variables in this study were derived from three levels of the ecological model (individual, social and physical environment) [27]. Children’s height (m) and weight (kg) were measured using standardized measurement procedures by trained researchers with a Wedderburn Seca portable rigid stadiometer and Wedderburn Tanita portable digital scales respectively [28, 29]. Parents self-reported their height and weight and that of their partner (where applicable); BMI was calculated (kg/m^2^) for children and parents. Child BMI categories were determined using age- and sex-specific international cut-off points [30, 31] and WHO classifications [32] were used for parents.

Parents reported individual domain correlates (*n* = 29) including biological and demographic variables (e.g., parent’s age, country of birth, education; child’s sleep duration, number of siblings); child behavioural variables (e.g., participation in organized activities, outdoor play time); and psychological variables (e.g., child preferences for physical activity and screen time). Social domain correlates (*n* = 26) included parental variables (e.g., parental constraints to supporting physical activity, parental rules and regulations regarding physical activity and screen time) and broader social variables (e.g., role-modelling of physical activity and screen time, social gatherings). Physical environment domain correlates (*n* = 12) included home environment variables (e.g., number of televisions in the home, indoor play spaces), and broader neighbourhood variables (e.g., park and playground availability and quality, frequency of visits to active play spaces). Only survey items with established test-retest reliability were included in analyses: for categorical items Kappa >0.60 and/or per cent agreement >60%; and for continuous variables ICC >0.50 [33]. See (Additional file 1: Table S1) for a full list of potential correlates included in analyses.

### Data analysis

Analyses were performed in Stata 13.0 (StataCorp, Texas, USA). Descriptive statistics were used to characterize the sample and t-tests were used to determine differences in sedentary and screen time between boys and girls. Multivariable linear regression models were used to identify correlates of sedentary time and screen time. Initially, each potential correlate was included in individual models with each of the two outcomes. Variables that were significant in individual models (*p* < 0.05) were included in combined models. Collinearity of variables included in the combined models was tested using tolerance and variance inflation factors (VIFs); no issues with collinearity were identified. Given that child age was positively associated with sedentary time for both boys (β = 8.33, 95% CI 4.46, 12.20) and girls (β = 7.93, 95% CI 2.78, 13.07), all models controlled for child age. Additionally, given that in Australian children have varying preschool/childcare hours, all models controlled for hours of preschool/childcare attendance per week. Models also controlled for clustering by centre of recruitment and were performed separately by child sex.

## Results

Descriptive data have previously been reported [8, 20]. Respondent parents (93.7% female) had a mean (SD) age of 37.3 (5.2) years and 69.8% were born in Australia. Children had a mean (SD) age of 4.5 (0.7) years. Boys spent a mean (SD) of 109.8 (69.8) minutes per day and girls spent a mean (SD) of 107.0 (69.4) minutes per day in screen time (*p* > 0.05). Accelerometry data showed that boys were sedentary for a mean (SD) of 303.0 (34.6) minutes per day while girls spent a mean (SD) of 298.8 (33.4) minutes per day sedentary (*p* > 0.05).

Table 1 shows individual model results for screen and sedentary time, stratified by child sex. Individual models showed that 22 variables (six, 11 and five from the individual, social and physical environment domains, respectively) were significantly associated with boys’ screen time, while 28 variables (10, 12 and six from the individual, social and physical environment domains, respectively) were significantly associated with girls’ screen time. For sedentary time, five potential correlates (two, one and two from the individual, social and physical environment domains, respectively) were identified in the individual models for boys and five (four, zero and one from the individual, social and physical environment domains, respectively) were identified for girls.

**Table 1:** Individual regression models^a^ for all potential correlates of screen time and sedentary time for boys and girls

Variable	Screen time mins/day				Sedentary time mins/day			
	Boys (*n* = 504)		Girls (*n* = 433)		Boys (*n* = 394)		Girls (*n* = 323)	
	% or mean (SD)^b^	β (95% CI)	% or mean (SD)^b^	β (95% CI)	% or mean (SD)^b^	β (95% CI)	% or mean (SD)^b^	β (95% CI)
Individual level								
*Demographic and family profile*								
Child disability/poor health	10.3%	4.57 (−17.79, 26.93)	5.6%	39.18 (−4.82, 83.18)	9.9%	−10.93 (−21.99, 0.12)	5.6%	**14.38 (0.64, 28.13)**
Child’s birth parents live together	86.9%	−5.90 (−30.42, 18.63)	89.3%	−10.91 (−40.22, 18.40)	88.1%	9.71 (−0.63, 20.05)	90.0%	−1.60 (−14.50, 11.30)
Child sleep duration (hours)	11.1 (1.1)	**−13.96 (−22.40, −5.52)**	11.0 (1.1)	**−10.24 (−16.91, −3.57)**	11.1 (1.0)	**−7.09 (−11.35, −2.82)**	11.0 (1.1)	**−6.52 (−9.20, −3.84)**
Child has siblings	84.9%	−0.98 (−17.83, 15.88)	82.3%	−8.30 (−24.40, 7.81)	86.6%	−4.63 (−16.17, 6.91)	83.5%	1.79 (−9.37, 12.96)
Child BMI category^c^								
Underweight/healthy weight (ref)	82.5%	0	80.8%	0	83.3%	0	80.0%	0
Overweight/obese	17.5%	2.64 (−14.22, 19.51)	19.2%	2.19 (−14.84, 19.21)	16.7%	−1.30 (−12.00, 10.02)	20.0%	6.94 (−3.84, 17.72)
Maternal age (years)	37.1 (5.3)	−0.13 (−1.65, 1.40)	37.1 (5.1)	−1.22 (−2.53, 0.09)	37.0 (5.1)	0.57 (−0.06, 1.21)	37.1 (5.2)	−0.30 (−1.07, 0.48)
Mother born in Australia	70.1%	**−17.00 (−32.69, −1.32)**	68.1%	**−26.60 (−42.88, −10.32)**	72.6%	−7.75 (−16.18, 0.69)	68.1%	−0.61 (−8.89, 7.67)
Maternal BMI category^d^								
Healthy weight (ref)	62.1%	0	60.9%	0	62.1%	0	62.8%	0
Overweight	22.7%	11.13 (−1.70, 23.96)	21.8%	7.28 (−8.60, 23.16)	23.4%	5.69 (−2.98, 14.37)	19.2%	**9.99 (0.51, 19.47)**
Obese	15.2%	17.04 (−8.42, 42.49)	17.3%	**26.78 (7.91, 45.65)**	14.6%	−8.80 (−18.27, 0.68)	18.0%	7.98 (−1.32, 17.28)
Mother in paid employment^e^	55.3%	−6.29 (−20.24, 7.66)	52.5%	−9.49 (−22.72, 3.74)	55.5%	5.63 (−2.12, 13.38)	50.8%	3.91 (−2.83, 10.65)
Maternal disability/poor health	3.4%	7.72 (−28.30, 43.75)	4.4%	33.04 (−3.77, 69.86)	2.5%	−17.05 (−35.42, 1.32)	4.6%	**19.32 (5.72, 32.92)**
Maternal education								
Year 10 or equivalent (ref)	10.6%	0	11.2%	0	9.9%	0	11.2%	0
Year 12/trade/diploma	31.7%	−6.72 (−34.83, 21.38)	36.3%	−10.37 (−34.49, 13.75)	31.0%	−7.21 (−21.03, 6.61)	34.7%	6.71 (−9.16, 22.57)
University degree/post-graduate	57.7%	−17.11 (−45.00, 10.78)	52.6%	**−34.98 (−57.46, −12.49)**	59.0%	−1.83 (−13.93, 10.28)	54.2%	−6.23 (−18.96, 6.49)
Low income status (health care/pension card)	20.4%	13.72 (−4.88, 32.33)	18.1%	**23.91 (3.92, 43.90)**	17.7%	−4.05 (−14.67, 6.57)	17.7%	4.40 (−5.70, 14.5)
Paternal age (years)	39.1 (5.7)	−0.65 (−1.74, 0.43)	39.2 (5.5)	−0.58 (−1.99, 0.83)	38.9 (5.5)	**0.85 (0.21, 1.50)**	39.0 (5.3)	−0.27 (−1.14, 0.59)
Father born in Australia	68.3%	−8.24 (−22.90, 6.42)	66.9%	**−19.14 (−35.97, −2.32)**	71.3%	−6.46 (−15.31, 2.39)	69.5%	−2.25 (−11.36, 6.86)
Paternal BMI category^d^								
Healthy weight (ref)	38.6%	0	34.0%	0	36.7%	0	36.5%	0
Overweight	45.2%	1.91 (−11.26, 15.08)	48.7%	4.62 (−11.62, 20.87)	46.9%	−0.38 (−9.41, 8.66)	47.0%	−3.47 (−11.23, 4.28)
Obese	16.2%	**18.84 (2.21, 35.48)**	17.3%	18.97 (−2.31, 40.24)	16.3%	8.57 (−2.73, 19.86)	16.5%	6.99 (−4.81, 18.80)
Father in paid employment^e^	88.9%	10.16 (−7.81, 28.13)	90.8%	−16.27 (−42.18, 9.64)	89.7%	1.96 (−11.21, 15.12)	90.9%	−10.51 (−27.72, 6.71)
Paternal disability/poor health	4.9%	32.13 (−6.56, 70.83)	4.4%	38.40 (−10.43, 87.23)	4.5%	14.39 (−3.29, 32.08)	4.8%	4.17 (−9.92, 18.26)
Paternal education								
Year 10 or equivalent (ref)	9.6%	0	9.5%	0	9.9%	0	7.8%	0
Year 12/trade/diploma	39.9%	−8.63 (−29.07, 11.82)	42.4%	0.26 (−25.22, 25.74)	39.2%	−4.91 (−16.47, 6.65)	41.8%	−8.10 (−26.21, 10.01)
University degree/post-graduate	50.5%	−14.67 (−33.75, 4.42)	48.1%	**−28.92 (−52.31, −5.53)**	51.0%	0.95 (−9.83, 11.73)	50.3%	−9.01 (−25.08, 7.06)
*Child PA and SB*								
Usual frequency of active transport per week (e.g., ride a bike to kinder)	4.6 (3.9)	−0.22 (−1.79, 1.35)	4.3 (4.1)	**−3.01 (−4.53, −1.49)**	4.4 (3.6)	−0.73 (−1.65, 0.19)	4.2 (3.9)	−0.10 (−1.28, 1.07)
Usual frequency of non-organised activities per week (e.g., play in the backyard)	22.7 (9.8)	−0.51 (−1.20, 0.19)	22.8 (10.4)	−0.35 (−0.80, 0.09)	22.3 (9.7)	−0.28 (−0.65, 0.09)	22.3 (10.4)	−0.18 (−0.56, 0.19)
Number of organised activities per week (e.g., swimming, tennis)	0.9 (0.8)	−3.75 (−12.14, 4.63)	1.2 (0.8)	**−17.67 (−25.95, −9.39)**	0.9 (0.8)	0.93 (−3.81, 5.67)	1.2 (0.8)	−3.29 (−7.79, 1.21)
Child attends playgroup^f^	24.3%	4.98 (−8.96, 18.92)	20.8%	1.52 (−16.25, 19.30)	24.1%	0.27 (−7.36, 7.90)	20.1%	−3.88 (−11.64, 3.88)
Average outdoor play time hours/day (week and weekend day)	4.3 (2.7)	0.11 (−2.60, 2.82)	3.8 (2.5)	1.18 (−1.27, 3.63)	4.2 (2.6)	−0.27 (−1.77, 1.24)	3.7 (2.5)	−0.75 (−2.33, 0.83)
*Child personality, preferences and constraints*								
Child active co-participation preferences (e.g., active by him/herself, active with his/her friends)	1.8 (1.5)	0.42 (−2.82, 3.65)	1.6 (1.4)	−0.78 (−5.74, 4.19)	1.8 (1.5)	−0.59 (−3.43, 2.25)	1.5 (1.4)	−1.92 (−4.60, 0.77)
Child is active for longer with someone else	79.2%	**−20.57 (−40.06, −1.08)**	78.3%	14.43 (−2.48, 31.34)	78.9%	−6.32 (−16.18, 3.55)	78.5%	−1.70 (−11.41, 8.00)
Child is competitive with other children when being active	66.2%	11.48 (−1.00, 23.96)	51.9%	−1.21 (−14.28, 11.86)	66.9%	2.75 (−3.41, 8.90)	51.3%	0.33 (−7.44, 8.09)
Child prosocial PA behaviour (e.g., asks for opportunities to be active)	12.8 (2.8)	0.29 (−2.17, 2.76)	12.6 (2.9)	−0.43 (−2.58, 1.73)	12.8 (2.7)	−0.24 (−1.40, 0.92)	12.5 (2.8)	−0.01 (−1.60, 1.58)
Child preferences for SB (e.g., more likely to watch TV than be active)	4.1 (1.6)	**10.07 (5.98, 14.16)**	4.4 (1.4)	**14.51 (9.71, 19.32)**	4.1 (1.6)	0.89 (−1.42, 3.20)	4.5 (1.4)	1.08 (−2.37, 4.53)
Child constraints to PA (e.g., too tired to do more PA)	−12.1 (4.5)	**1.60 (0.11, 3.09)**	−11.9 (4.4)	1.28 (−0.07, 2.63)	−12.2 (4.4)	−0.48 (−1.21, 0.25)	−11.8 (4.5)	−0.33 (−1.12, 0.46)
Social level								
*Parental influence*								
Parental concerns about child’s PA/SB	-3.9 (3.3)	**4.59 (2.51, 6.66)**	−4.4 (3.1)	**7.47 (4.90, 10.04)**	−3.8 (3.3)	0.57 (−0.44, 1.57)	−4.3 (3.0)	0.59 (−0.69, 1.87)
Parental constraints to child’s PA	6.9 (3.1)	**2.87 (0.36, 5.38)**	6.7 (3.2)	**5.05 (3.19, 6.92)**	6.9 (3.0)	0.14 (−1.01, 1.28)	6.8 (3.1)	0.94 (−0.19, 2.07)
Parent likes to participate in outdoor play	73.5%	4.70 (−8.72, 18.12)	70.8%	7.03 (−7.35, 21.41)	73.0%	3.65 (−3.37, 10.67)	70.0%	−5.31 (−13.37, 2.75)
Parent prefers to be social with other parents	36.7%	−5.65 (−17.2, 5.90)	39.1%	11.65 (−1.77, 25.07)	35.6%	−0.68 (−7.52, 6.15)	36.8%	−2.02 (−10.21, 6.16)
Parent gets bored watching child playing in outdoor spaces	11.8%	**−22.01 (−36.87, −7.15)**	12.5%	5.81 (−18.58, 30.19)	11.5%	−0.71 (−8.44, 7.02)	12.7%	−7.79 (−18.44, 2.86)
Parent likes child to do activities of older children	24.0%	−10.26 (−24.05, 3.52)	22.5%	12.73 (−2.27, 27.73)	24.7%	1.18 (−6.7, 9.07)	22.0%	6.29 (−2.93, 15.50)
Parent likes child to do activities they did as a child	34.3%	5.27 (−9.72, 20.26)	33.0%	1.79 (−12.19, 15.76)	34.4%	−0.71 (−7.41, 5.99)	34.1%	−0.01 (−8.59, 8.58)
Parent gets bored going to the same place	12.8%	5.71 (−21.47, 32.88)	10.9%	7.73 (−17.83, 33.29)	10.7%	−0.83 (−10.72, 9.06)	11.2%	−6.60 (−16.29, 3.09)
Parent believes it’s important to be active as a family	90.8%	**−26.67 (−48.93, −4.42)**	86.9%	−14.56 (−34.31, 5.19)	90.6%	−0.22 (−12.23, 11.80)	86.8%	−6.87 (−19.39, 5.65)
Parental self-efficacy to support PA	5.9 (1.7)	**−3.62 (−7.02, −0.23)**	5.9 (1.8)	**−8.07 (−11.75, −4.38)**	5.9 (1.7)	0.06 (−2.00, 2.12)	5.8 (1.8)	−0.54 (−2.60, 1.53)
Parental self-efficacy to limit screen time	8.7 (2.3)	**−9.15 (−11.68, −6.62)**	8.9 (2.5)	**−8.25 (−10.98, −5.52)**	8.8 (2.3)	−0.20 (−1.86, 1.46)	9.0 (2.4)	−0.89 (−2.43, 0.66)
Parental health knowledge/beliefs of child’s PA	1.5 (2.2)	−4.19 (−8.74, 0.36)	1.3 (2.2)	**−5.65 (−8.87, −2.44)**	1.6 (2.1)	−0.08 (−1.72, 1.56)	1.3 (2.2)	0.46 (−1.31, 2.23)
*Rules and boundaries*								
Parental rules to limit screen time	2.1 (1.6)	**−11.40 (−16.32, −6.48)**	2.2 (1.5)	**−14.20 (−18.24, −10.16)**	2.2 (1.6)	−0.64 (−2.65, 1.37)	2.2 (1.5)	−1.90 (−4.46, 0.66)
Parental rules about games inside (e.g., no throwing balls inside)	−0.2 (2.1)	1.13 (−2.50, 4.76)	−0.2 (2.1)	1.13 (−2.50, 4.76)	−0.2 (2.1)	−0.16 (−1.90, 1.58)	−0.1 (2.1)	1.16 (−0.73, 3.05)
Parental rules about PA for stranger danger, traffic, injury	2.3 (1.5)	−1.23 (−6.15, 3.70)	2.3 (1.6)	1.27 (−2.65, 5.19)	2.3 (1.5)	−1.73 (−4.17, 0.71)	2.3 (1.5)	−0.31 (−3.17, 2.54)
Parent allows child to play freely in backyard/street	−0.1 (1.2)	−6.29 (−13.74, 1.16)	−0.1 (1.2)	**−7.11 (−12.49, −1.73)**	−0.1 (1.1)	−2.67 (−6.09, 0.75)	−0.04 (1.2)	−0.97 (−3.99, 2.05)
Parent switches off screen entertainment	2.7 (1.4)	3.19 (−0.62, 7.00)	2.6 (1.3)	3.26 (−1.76, 8.28)	2.7 (1.4)	−0.27 (−2.80, 2.26)	2.7 (1.3)	−0.83 (−3.35, 1.69)
*Social interaction and support*								
Child is active at social gatherings	6.0 (1.0)	5.26 (−1.42, 11.95)	6.0 (1.2)	1.05 (−5.88, 7.97)	6.0 (1.0)	−0.54 (−5.45, 4.37)	5.9 (1.2)	−1.86 (−4.50, 0.77)
Maternal PA emotional support for child	5.5 (2.1)	**4.32 (1.74, 6.90)**	5.4 (2.1)	1.20 (−1.84, 4.24)	5.5 (2.1)	**−1.75 (−3.46, −0.04)**	5.4 (2.1)	−0.92 (−2.55, 0.71)
Paternal PA emotional support for child	4.7 (2.4)	**4.29 (1.89, 6.70)**	4.6 (2.4)	1.86 (−0.95, 4.66)	4.7 (2.4)	−1.25 (−2.77, 0.28)	4.6 (2.4)	−0.73 (−2.43, 0.97)
*Modelling of PA*								
Maternal time in PA (hours/week)	5.4 (4.2)	−0.01 (−1.72, 1.69)	5.0 (4.0)	−1.28 (−3.01, 0.45)	5.3 (4.0)	−0.17 (−0.79, 0.45)	4.9 (3.9)	0.46 (−0.54, 1.45)
Paternal time in PA (hours/week)	5.4 (4.7)	0.24 (−0.93, 1.40)	5.2 (4.6)	**−2.12 (−3.65, −0.59)**	5.0 (4.3)	−0.47 (−1.17, 0.22)	5.2 (4.5)	0.35 (−0.53, 1.23)
Maternal TV viewing (hours/week)	8.6 (6.8)	**3.87 (1.81, 5.93)**	9.0 (6.5)	**2.62 (1.56, 3.68)**	8.6 (6.1)	−0.14 (−0.74, 0.46)	9.2 (6.6)	0.10 (−0.46, 0.66)
Paternal TV viewing (hours/week)	9.6 (6.6)	**1.91 (0.96, 2.85)**	9.8 (7.0)	**2.26 (1.23, 3.29)**	9.8 (6.4)	0.16 (−0.39, 0.71)	9.6 (6.9)	−0.01 (−0.48, 0.46)
Maternal role modelling of PA (times/week)	3.2 (2.0)	−0.63 (−4.49, 3.23)	3.0 (2.0)	**−3.67 (−6.97, −0.38)**	3.3 (2.0)	−0.29 (−1.94, 1.35)	3.0 (2.0)	−1.03 (−3.27, 1.22)
Paternal role modelling of PA (times/week)	2.8 (2.0)	−1.82 (−4.88, 1.24)	2.6 (2.0)	**−4.68 (−7.73, −1.63)**	2.8 (1.9)	−0.21 (−2.01, 1.60)	2.7 (2.0)	−0.28 (−2.26, 1.69)
Physical environment level								
Dog ownership	32.3%	3.35 (−9.64, 16.34)	32.2%	−1.44 (−14.89, 12.00)	32.1%	0.90 (−6.43, 8.22)	31.2%	4.94 (−2.91, 12.79)
Number of pieces of toys/ equipment to be physically active with at home (e.g., swings, slide)	13.3 (3.5)	−1.62 (−3.25, 0.001)	13.1 (3.7)	**−2.37 (−4.15, −0.60)**	13.4 (3.5)	−0.12 (−1.17, 0.93)	13.1 (3.7)	−0.69 (−1.89, 0.52)
Lives on medium/large block	86.1%	1.16 (−14.99, 17.30)	85.2%	−8.11 (−26.65, 10.43)	86.1%	−1.06 (−12.25, 10.13)	84.5%	−6.63 (−18.48, 5.21)
Number of features at home (e.g., front fence, covered outdoor areas)	1.9 (0.9)	**−11.00 (−20.38, −1.62)**	1.9 (0.9)	**−11.48 (−17.82, −5.14)**	2.0 (0.8)	2.02 (−2.10, 6.14)	1.9 (0.8)	−3.14 (−8.23, 1.95)
Lives on a cul-de-sac	24.1%	**24.23 (7.27, 41.19)**	29.6%	13.57 (−1.66, 28.79)	23.3%	−5.76 (−13.49, 1.97)	28.0%	0.31 (−7.40, 8.01)
Number of pieces of electronic equipment at home (e.g., DVD player, PlayStation)	5.1 (1.2)	3.83 (−2.76, 10.41)	5.1 (1.1)	2.89 (−2.67, 8.46)	5.1 (1.1)	1.01 (−2.89, 4.91)	5.0 (1.1)	1.58 (−2.33, 5.49)
Number of TVs at home	2.2 (1.2)	**7.31 (1.89, 12.74)**	2.2 (1.1)	**14.08 (7.61, 20.55)**	2.2 (1.1)	−0.80 (−3.60, 2.00)	2.2 (1.0)	**4.10 (1.00, 7.20)**
TV in child’s bedroom	10.6%	**23.51 (4.75, 42.28)**	9.5%	**50.01 (23.84, 76.18)**	10.0%	0.02 (−11.97, 12.01)	7.1%	12.36 (−1.91, 26.63)
Computer/e-games in child’s bedroom	4.2%	11.34 (−28.46, 51.15)	4.2%	5.86 (−22.70, 34.41)	4.1%	17.37 (−9.11, 43.85)	3.7%	15.80 (−1.21, 32.81)
Neighbourhood playground suitability (e.g., equipment, shade, safety)	5.5 (4.4)	**−2.98 (−5.30, −0.66)**	5.2 (4.4)	**−2.71 (−4.15, −1.28)**	5.6 (4.2)	**−0.92 (−1.65, −0.19)**	5.5 (4.5)	−0.31 (−1.13, 0.52)
Neighbourhood constraints to active transport (e.g., busy roads)	4.9 (4.4)	−1.46 (−3.19, 0.28)	4.8 (4.6)	**−2.06 (−3.50, −0.62)**	5.0 (4.4)	**−0.65 (−1.28, −0.01)**	5.0 (4.4)	−0.42 (−1.34, 0.50)
Total frequency of visiting active places per week	6.5 (3.2)	0.64 (−1.58, 2.85)	5.9 (3.0)	0.24 (−2.62, 3.10)	6.4 (3.2)	−0.77 (−1.90, 0.37)	5.9 (3.1)	−0.35 (−1.60, 0.90)

*Abbreviations: BMI* body mass index, *CI* confidence interval, *e-games* electronic games, *PA* physical activity, *SB* sedentary behaviour, *TV* television

^a^All models adjusted for age, preschool/childcare attendance and clustering by centre of recruitment; ^b^ Reported as % for binary/categorical variables and mean (SD) for continuous variables; ^c^ Directly measured height and weight, calculated using Cole et al. classifications; ^d^ Parents’ self-reported height and weight, calculated using WHO classifications; ^e^ Includes part- and full-time paid employment; ^f^ In Australia, playgroups are informal gatherings for parents (and caregivers) and their children prior to the commencement of school; bolded data indicates significance (*p* < 0.05); **−** indicates variable not included in combined model

Table 2 presents the results of the combined models for correlates of screen and sedentary time, stratified by child sex. In the combined model, for boys, five correlates remained significantly associated with screen time and one correlate remained significantly associated with sedentary time. For girls, seven correlates remained significantly associated with screen time and one variable remained significantly associated with sedentary time. No common correlates of screen and sedentary time were identified for either boys or girls.

**Table 2:** Combined regression models^a^ for correlates of screen time and sedentary time for boys and girls

Variable	Screen time mins/day		Sedentary time mins/day	
	Boys (*n* = 504)	Girls (*n* = 433)	Boys (*n* = 394)	Girls (*n* = 323)
	β (95% CI)	β (95% CI)	β (95% CI)	β (95% CI)
Individual level				
*Demographic and family profile*				
Child disability/poor health	-	-	-	10.18 (−3.03, 23.40)
Child sleep duration (hours)	**−7.49 (−13.46, −1.52)**	−5.67 (−11.57, 0.23)	−3.97 (−7.95, 0.01)	**−5.76 (−8.83, −2.69)**
Mother born in Australia	0.99 (−11.45, 13.42)	**−15.66 (−28.97, −2.35)**	-	-
Maternal BMI category^c^				
Healthy weight (ref)	-	0	-	0
Overweight	-	1.56 (−12.22, 15.34)	-	8.55 (−0.90, 18.00)
Obese	-	15.11 (−4.11, 34.32)	-	5.20 (−4.05, 14.45)
Maternal disability/poor health	-	-	-	10.97 (−5.36, 27.30)
Maternal education				
Year 10 or equivalent (ref)	-	0	-	-
Year 12/trade/diploma	-	−3.39 (−27.86, 21.09)	-	-
University degree/post-graduate	-	−2.62 (−30.91, 25.66)	-	-
Low income status (health care/pension card)	-	−4.22 (−23.18, 14.74)	-	-
Paternal age (years)	-	-	**0.73 (0.10, 1.35)**	-
Father born in Australia	-	−3.93 (−17.82, 9.95)	-	-
Paternal BMI category^c^				
Healthy weight (ref)	0	-	-	-
Overweight	−1.15 (−13.03, 10.74)	-	-	-
Obese	2.79 (−12.80, 18.37)	-	-	-
Paternal education				
Year 10 or equivalent (ref)	-	0	-	-
Year 12/trade/diploma	-	**23.26 (0.11, 46.41)**	-	-
University degree/post-graduate	-	9.66 (−16.33, 35.65)	-	-
*Child PA and SB*				
Usual frequency of active transport per week (e.g., ride a bike to kinder)	-	**−1.72 (−3.14, −0.30)**	-	-
Number of organised activities per week (e.g., swimming, tennis)	-	−7.54 (−15.72, 0.64)	-	-
*Child personality, preferences and constraints*				
Child is active for longer with someone else	−10.63 (−23.18, 1.93)	-	-	-
Child preferences for SB (e.g., more likely to watch TV than be active)	3.52 (−1.49, 8.53)	**7.09 (2.47, 11.70)**	-	-
Child constraints to PA (e.g., too tired to do more PA)	−0.69 (−2.12, 0.75)	-	-	-
Social level				
*Parental influence*				
Parental concerns about child’s PA/SB	1.87 (−0.05, 3.78)	**3.19 (0.62, 5.76)**	-	-
Parental constraints to child’s PA	0.74 (−1.36, 2.84)	1.29 (−0.55, 3.13)	-	-
Parent gets bored watching child playing in outdoor spaces	**−14.43 (−27.99, −0.86)**	-	-	-
Parent believes it’s important to be active as a family	6.08 (−10.86, 23.02)	-	-	-
Parental self-efficacy to support PA	0.76 (−2.78, 4.30)	1.67 (−2.23, 5.58)	-	-
Parental self-efficacy to limit screen time	**−6.52 (−9.51, −3.54)**	**−2.64 (−5.12, −0.16)**	-	-
Parental health knowledge/beliefs of child’s PA	-	0.22 (−3.67, 4.11)	-	-
*Rules and boundaries*				
Parental rules to limit screen time	**−5.15 (−9.20, −1.11)**	**−5.20 (−9.94, −0.47)**	-	-
Parent allows child to play freely in backyard/street	-	−4.62 (−9.39, 0.16)	-	-
***Social interaction and support***				
Maternal PA emotional support for child	2.14 (−1.47, 5.76)	-	−1.10 (−2.84, 0.63)	-
Paternal PA emotional support for child	2.14 (−0.87, 5.14)	-	-	-
*Modelling of PA*				
Paternal time in PA (hours/week)	-	−0.62 (−2.29, 1.04)	-	-
Maternal TV viewing (hours/week)	**2.27 (1.09, 3.46)**	1.33 (−0.05, 2.70)	-	-
Paternal TV viewing (hours/week)	0.49 (−0.71, 1.69)	0.65 (−0.33, 1.63)	-	-
Maternal role modelling of PA (times/week)	-	0.36 (−3.27, 4.00)	-	-
Paternal role modelling of PA (times/week)	-	0.54 (−3.54, 4.61)	-	-
Physical environment level				
Number of pieces of toys/ equipment to be physically active with at home (e.g., swings, slide)	-	0.57 (−1.15, 2.29)	-	-
Number of features at home (e.g., front fence, covered outdoor areas)	−2.51 (−9.95, 4.92)	−2.34 (−9.40, 4.72)	-	-
Lives on a cul-de-sac	11.70 (−1.26, 24.66)	-	-	-
Number of TVs at home	3.65 (−2.04, 9.34)	5.15 (−1.69, 11.99)	-	2.45 (−0.79, 5.70)
TV in child’s bedroom	−1.74 (−23.72, 20.23)	27.14 (−5.49, 59.77)	-	-
Neighbourhood playground suitability (e.g., equipment, shade, safety)	0.11 (−1.10, 1.32)	−0.69 (−2.11, 0.73)	−0.69 (−1.51, 0.12)	-
Neighbourhood constraints to active transport (e.g., busy roads)	-	0.14 (−1.29, 1.57)	−0.26 (−0.96, 0.43)	-

*Abbreviations*: *BMI* body mass index, *CI* confidence interval, *PA* physical activity, *SB* sedentary behaviour, *TV* television

^a^ All models adjusted for age, preschool/childcare attendance and clustering by centre of recruitment; ^b^ Reported as % for binary/categorical variables and mean (SD) for continuous variables; ^c^ Parents’ self-reported height and weight, calculated using WHO classifications; bolded data indicates significance (*p* < 0.05); **−** indicates variable not included in combined model

In the combined model, for each additional hour of sleep, boys spent 7.5 min less per day in screen time. For every unit increase in parental self-efficacy to limit screen time and their actual rules to limit screen time, boys spent 6.5 min and 5.2 min less per day in screen time, respectively. If parents reported that they get bored watching their child play, boys spent 14.4 min less per day in screen time. Conversely, maternal television viewing was positively associated with boys’ screen time, such that boys spent an additional 2.3 min per day in screen time for each additional hour in maternal television viewing. For sedentary time, boys spent an additional 0.7 min per day sedentary for every additional year of paternal age.

For girls, results from the combined model show that if mothers were born in Australia, girls spent 15.7 min less per day in screen time. For every unit increase in parental self-efficacy to limit screen time and rules to limit screen time, girls spent 6.5 min and 2.6 min less per day in screen time, respectively. Paternal education, child preferences for sedentary behaviour (e.g. child is more likely to watch television than be active), and parental concerns about their child’s physical activity and sedentary behaviour were positively associated with girls’ screen time. If fathers had a year 12/trade/diploma level of education, girls spent 23.3 min more per day in screen time compared to fathers with a year 10 or equivalent level of education. Girls also spent 7.1 min per day more in screen time for each unit increase in parent-reported child preferences for sedentary behaviour, and 3.1 min more per day in screen time for every unit increase in parental concerns about their child’s physical activity and sedentary behaviour. For sedentary time, girls spent 5.8 min less per day sedentary for every additional hour of sleep time.

## Discussion

The aim of this study was to identify whether correlates of screen time and sedentary time in preschool children differ. Results identified a greater number of correlates of screen time than sedentary time in this population. No common correlates of screen and sedentary time were identified for either boys or girls. The larger number of correlates of screen time than sedentary time is consistent with research in older children [17, 18]. This may be because in this study both screen time and potential correlates were parent-reported, whereas sedentary time was objectively measured, hence there may have been consistent reporting biases that influenced associations for screen time. Additionally, many of the correlates measured focus directly on screen time (e.g., parents limiting screen time) rather than sedentary time (e.g., strategies to reduce overall sitting). They may therefore be less relevant to sedentary time which, when measured by accelerometry, captures many more types of sedentary behaviour in addition to screens (e.g., reading, craft, quiet play) across many domains (e.g., in the car, at preschool, in the home). Most research to date has focused only on screen time [14], which is often used as a proxy for sedentary time [34]. However, results from the current study suggest that the correlates of these behaviours differ, and therefore behaviour-specific strategies may be required to reduce screen time and sedentary time.

Children’s total sleep time (including daytime naps) was significantly inversely associated with girls’ sedentary time and boys’ screen time. The association between sedentary time and sleep has not previously been investigated in preschool children, but research in older children supports this inverse association [17]. Previous research has found that increased sleep time is associated with decreased television viewing in five-year-old children [35]. In the current study it is not possible to determine whether children are engaging in higher levels of screen time and sedentary time due to less sleep, or whether the higher levels of sedentary time and screen time disrupt sleep. However, screen time at age two years has been longitudinally inversely associated with sleep duration at age five years [36], suggesting that encouraging parents to decrease their child’s screen time to improve sleep could be an appealing strategy for parents.

The only other common correlates for boys’ and girls’ screen time were parental self-efficacy to limit screen time and their actual rules to limit screen time. For every unit increase in the summed score for parental self-efficacy to limit screen time, boys and girls spent around six and three minutes less per day in screen time, respectively. Similarly, for every unit increase in parental rules, boys and girls both spent around five minutes less per day in screen time. Parental self-efficacy to limit screen time and their actual rules have consistently been shown to be inversely associated with screen time in preschool children [37–43]. This suggests that interventions and public health strategies to reduce sedentary behaviour could potentially give parents strategies to implement screen time rules, and in turn increase parental self-efficacy to limit screen time.

Consistent with research in school-aged children [17], there were a higher number of parent demographic correlates of girls’ compared to boys’ screen time: maternal ethnicity and paternal education were both associated with girls’ screen time, while there were no parent demographic correlates associated with boys’ screen time. Conversely, there were a larger number of parental influences in the social level of the ecological model associated with boys’ compared to girls’ screen time. If parents reported that they get bored watching their child play in outdoor spaces, boys spent around 14 min less per day in screen time. It may be that these children have higher levels of physical activity (and therefore potentially lower levels of screen time) so their parents get bored watching for long periods of time.

Many of the associations identified in this study were relatively modest in magnitude e.g., a seven minutes less screen time for every additional hour of sleep. However, when considered in light of screen time recommendations for this age group (i.e., one hour or less per day), seven minutes equates to around 12% of this time. Given that there is evidence of a dose-response for increased screen time and poorer cognitive development and psychological health [4], even modest decreases in screen time may have significant health benefits in early childhood. Additionally, it is important to note that the magnitude of associations seen in the current study are the average for the sample, but across the population may be important for public health.

Consistent with previous research [15, 19], the current study found that the correlates of both screen time and sedentary time differ between boys and girls. These findings suggest that future research should recruit samples that are sufficiently large to ensure adequate power to stratify analyses by sex. Additionally, future interventions would benefit from using sex-specific strategies to reduce time in these behaviours. Despite these differences in correlates, results from this study show that preschool boys and girls spend similar amounts of time engaging in screen time and sedentary time. Previous reviews have consistently found that child sex is not associated with screen time in this population [13, 14, 44]. However, there is an indeterminate association between child sex and sedentary time, with some studies finding that preschool girls are more sedentary than boys [15, 45, 46] and others finding no association [47, 48]. Given that girls are consistently shown to be more sedentary than boys in research involving school-aged children and adolescents [49], it may be that the sex-difference in sedentary time increases as children age. This suggests that girls may particularly benefit from early intervention.

There were several strengths to this study including the use of accelerometers to objectively assess sedentary time. Additionally, this study included a wide range of potential correlates covering multiple domains of the ecological model, with the parent survey purpose-designed to cover these domains and tested for reliability [33]. Despite the low response rate (11%), the sample was large and recruited across low-, mid- and high-socioeconomic areas. Demographic characteristics were comparable with 2011 national census data; e.g., 70% of parents vs 70% of adults born in Australia, 67% of parents vs 58% of adults with post-secondary qualifications [50]. However, results may be specific to suburban Melbourne and may not be generalizable to rural areas or other cities or countries. The cross-sectional design of the study prohibits inference of causality; future studies would benefit from employing a longitudinal design to determine causality.

Future work would also benefit from including sedentary behaviours beyond just screen time. Currently, very little is known about other, non-screen based sedentary behaviours that may have positive physical, mental and cognitive health effects (e.g., reading, quiet play). Having a better understanding of the factors associated with these other types of sedentary behaviour would help inform public health messages and interventions to reduce time in unfavourable sedentary behaviours. The current study does identify a number of modifiable factors that are associated with both screen time and sedentary time in preschool children. In particular, parental factors such as self-efficacy, modelling, and screen time rules could be potential targets for future interventions.

## Conclusions

Contrary to public health recommendations, preschool children are spending large amounts of time engaging in screen and sedentary time. Few common correlates exist for screen time and sedentary time suggesting that different strategies to reduce screen time and sedentary time in this population are needed. Similarly, there were a number of different correlates for boys and girls, signifying that sex-specific strategies may be required to reduce sedentary behaviours. Parental correlates (such as self-efficacy and screen time rules) identified in this study are modifiable and could potentially be targeted in interventions and public health strategies to reduce sedentary behaviour in preschool children.

## Additional file

Additional file 1:
**Table S1.** Potential correlates of sedentary time and screen time included in individual models. (DOCX 22 kb)

## Abbreviations

BMI: Body mass index
CI: Confidence interval
E-games: Electronic games
HAPPY: Healthy active preschool and primary years
LGA: Local government area
MET: Metabolic equivalent of task
PA: Physical activity
SB: Sedentary behaviour
TV: Television
VIF: Variance inflation factor
