# Erratum to: Do the correlates of screen time and sedentary time differ in preschool children?

**DOI:** 10.1186/s12889-017-4276-x

**Published:** 2017-04-28

**Authors:** Katherine L Downing, Trina Hinkley, Jo Salmon, Jill A Hnatiuk, Kylie D Hesketh

**Affiliations:** 1 Institute for Physical Activity and Nutrition (IPAN), School of Exercise and Nutrition Sciences, Deakin University, Geelong, Australia; 2 School of Science and Health, Western Sydney University, Penrith, NSW 2751 Australia

## Erratum

Following publication of this article [1], it has come to our attention that the formatting in Tables 1 and 2 has affected the alignment in such a way that some of the data and categories are difficult to read. The original article has been updated and the correct versions of the tables can also be viewed below.

**Table 1:** Individual regression models^a^ for all potential correlates of screen time and sedentary time for boys and girls

Variable	Screen time mins/day				Sedentary time mins/day			
	Boys (*n*=504)		Girls (*n*=433)		Boys (*n*=394)		Girls (*n*=323)	
	% or mean (SD)^b^	β (95% CI)	% or mean (SD)^b^	β (95% CI)	% or mean (SD)^b^	β (95% CI)	% or mean (SD)^b^	β (95% CI)
**Individual level**								
*Demographic and family profile*								
Child disability/poor health	10.3%	4.57 (-17.79, 26.93)	5.6%	39.18 (-4.82, 83.18)	9.9%	-10.93 (-21.99, 0.12)	5.6%	**14.38 (0.64, 28.13)**
Child’s birth parents live together	86.9%	-5.90 (-30.42, 18.63)	89.3%	-10.91 (-40.22, 18.40)	88.1%	9.71 (-0.63, 20.05)	90.0%	-1.60 (-14.50, 11.30)
Child sleep duration (hours)	11.1 (1.1)	**-13.96 (-22.40, -5.52)**	11.0 (1.1)	**-10.24 (-16.91, -3.57)**	11.1 (1.0)	**-7.09 (-11.35, -2.82)**	11.0 (1.1)	**-6.52 (-9.20, -3.84)**
Child has siblings	84.9%	-0.98 (-17.83, 15.88)	82.3%	-8.30 (-24.40, 7.81)	86.6%	-4.63 (-16.17, 6.91)	83.5%	1.79 (-9.37, 12.96)
Child BMI category^c^								
Underweight/healthy weight (ref)	82.5%	0	80.8%	0	83.3%	0	80.0%	0
Overweight/obese	17.5%	2.64 (-14.22, 19.51)	19.2%	2.19 (-14.84, 19.21)	16.7%	-1.30 (-12.00, 10.02)	20.0%	6.94 (-3.84, 17.72)
Maternal age (years)	37.1 (5.3)	-0.13 (-1.65, 1.40)	37.1 (5.1)	-1.22 (-2.53, 0.09)	37.0 (5.1)	0.57 (-0.06, 1.21)	37.1 (5.2)	-0.30 (-1.07, 0.48)
Mother born in Australia	70.1%	**-17.00 (-32.69, -1.32)**	68.1%	**-26.60 (-42.88, -10.32)**	72.6%	-7.75 (-16.18, 0.69)	68.1%	-0.61 (-8.89, 7.67)
Maternal BMI category^d^								
Healthy weight (ref)	62.1%	0	60.9%	0	62.1%	0	62.8%	0
Overweight	22.7%	11.13 (-1.70, 23.96)	21.8%	7.28 (-8.60, 23.16)	23.4%	5.69 (-2.98, 14.37)	19.2%	**9.99 (0.51, 19.47)**
Obese	15.2%	17.04 (-8.42, 42.49)	17.3%	**26.78 (7.91, 45.65)**	14.6%	-8.80 (-18.27, 0.68)	18.0%	7.98 (-1.32, 17.28)
Mother in paid employment^e^	55.3%	-6.29 (-20.24, 7.66)	52.5%	-9.49 (-22.72, 3.74)	55.5%	5.63 (-2.12, 13.38)	50.8%	3.91 (-2.83, 10.65)
Maternal disability/poor health	3.4%	7.72 (-28.30, 43.75)	4.4%	33.04 (-3.77, 69.86)	2.5%	-17.05 (-35.42, 1.32)	4.6%	**19.32 (5.72, 32.92)**
Maternal education								
Year 10 or equivalent (ref)	10.6%	0	11.2%	0	9.9%	0	11.2%	0
Year 12/trade/diploma	31.7%	-6.72 (-34.83, 21.38)	36.3%	-10.37 (-34.49, 13.75)	31.0%	-7.21 (-21.03, 6.61)	34.7%	6.71 (-9.16, 22.57)
University degree/post-graduate	57.7%	-17.11 (-45.00, 10.78)	52.6%	**-34.98 (-57.46, -12.49)**	59.0%	-1.83 (-13.93, 10.28)	54.2%	-6.23 (-18.96, 6.49)
Low income status (health care/pension card)	20.4%	13.72 (-4.88, 32.33)	18.1%	**23.91 (3.92, 43.90)**	17.7%	-4.05 (-14.67, 6.57)	17.7%	4.40 (-5.70, 14.5)
Paternal age (years)	39.1 (5.7)	-0.65 (-1.74, 0.43)	39.2 (5.5)	-0.58 (-1.99, 0.83)	38.9 (5.5)	**0.85 (0.21, 1.50)**	39.0 (5.3)	-0.27 (-1.14, 0.59)
Father born in Australia	68.3%	-8.24 (-22.90, 6.42)	66.9%	**-19.14 (-35.97, -2.32)**	71.3%	-6.46 (-15.31, 2.39)	69.5%	-2.25 (-11.36, 6.86)
Paternal BMI category^d^								
Healthy weight (ref)	38.6%	0	34.0%	0	36.7%	0	36.5%	0
Overweight	45.2%	1.91 (-11.26, 15.08)	48.7%	4.62 (-11.62, 20.87)	46.9%	-0.38 (-9.41, 8.66)	47.0%	-3.47 (-11.23, 4.28)
Obese	16.2%	**18.84 (2.21, 35.48)**	17.3%	18.97 (-2.31, 40.24)	16.3%	8.57 (-2.73, 19.86)	16.5%	6.99 (-4.81, 18.80)
Father in paid employment^e^	88.9%	10.16 (-7.81, 28.13)	90.8%	-16.27 (-42.18, 9.64)	89.7%	1.96 (-11.21, 15.12)	90.9%	-10.51 (-27.72, 6.71)
Paternal disability/poor health	4.9%	32.13 (-6.56, 70.83)	4.4%	38.40 (-10.43, 87.23)	4.5%	14.39 (-3.29, 32.08)	4.8%	4.17 (-9.92, 18.26)
Paternal education								
Year 10 or equivalent (ref)	9.6%	0	9.5%	0	9.9%	0	7.8%	0
Year 12/trade/diploma	39.9%	-8.63 (-29.07, 11.82)	42.4%	0.26 (-25.22, 25.74)	39.2%	-4.91 (-16.47, 6.65)	41.8%	-8.10 (-26.21, 10.01)
University degree/post-graduate	50.5%	-14.67 (-33.75, 4.42)	48.1%	**-28.92 (-52.31, -5.53)**	51.0%	0.95 (-9.83, 11.73)	50.3%	-9.01 (-25.08, 7.06)
*Child PA and SB*								
Usual frequency of active transport per week (e.g., ride a bike to kinder)	4.6 (3.9)	-0.22 (-1.79, 1.35)	4.3 (4.1)	**-3.01 (-4.53, -1.49)**	4.4 (3.6)	-0.73 (-1.65, 0.19)	4.2 (3.9)	-0.10 (-1.28, 1.07)
Usual frequency of non-organised activities per week (e.g., play in the backyard)	22.7 (9.8)	-0.51 (-1.20, 0.19)	22.8 (10.4)	-0.35 (-0.80, 0.09)	22.3 (9.7)	-0.28 (-0.65, 0.09)	22.3 (10.4)	-0.18 (-0.56, 0.19)
Number of organised activities per week (e.g., swimming, tennis)	0.9 (0.8)	-3.75 (-12.14, 4.63)	1.2 (0.8)	**-17.67 (-25.95, -9.39)**	0.9 (0.8)	0.93 (-3.81, 5.67)	1.2 (0.8)	-3.29 (-7.79, 1.21)
Child attends playgroup^f^	24.3%	4.98 (-8.96, 18.92)	20.8%	1.52 (-16.25, 19.30)	24.1%	0.27 (-7.36, 7.90)	20.1%	-3.88 (-11.64, 3.88)
Average outdoor play time hours/day (week and weekend day)	4.3 (2.7)	0.11 (-2.60, 2.82)	3.8 (2.5)	1.18 (-1.27, 3.63)	4.2 (2.6)	-0.27 (-1.77, 1.24)	3.7 (2.5)	-0.75 (-2.33, 0.83)
*Child personality, preferences and constraints*								
Child active co-participation preferences (e.g., active by him/herself, active with his/her friends)	1.8 (1.5)	0.42 (-2.82, 3.65)	1.6 (1.4)	-0.78 (-5.74, 4.19)	1.8 (1.5)	-0.59 (-3.43, 2.25)	1.5 (1.4)	-1.92 (-4.60, 0.77)
Child is active for longer with someone else	79.2%	**-20.57 (-40.06, -1.08)**	78.3%	14.43 (-2.48, 31.34)	78.9%	-6.32 (-16.18, 3.55)	78.5%	-1.70 (-11.41, 8.00)
Child is competitive with other children when being active	66.2%	11.48 (-1.00, 23.96)	51.9%	-1.21 (-14.28, 11.86)	66.9%	2.75 (-3.41, 8.90)	51.3%	0.33 (-7.44, 8.09)
Child prosocial PA behaviour (e.g., asks for opportunities to be active)	12.8 (2.8)	0.29 (-2.17, 2.76)	12.6 (2.9)	-0.43 (-2.58, 1.73)	12.8 (2.7)	-0.24 (-1.40, 0.92)	12.5 (2.8)	-0.01 (-1.60, 1.58)
Child preferences for SB (e.g., more likely to watch TV than be active)	4.1 (1.6)	**10.07 (5.98, 14.16)**	4.4 (1.4)	**14.51 (9.71, 19.32)**	4.1 (1.6)	0.89 (-1.42, 3.20)	4.5 (1.4)	1.08 (-2.37, 4.53)
Child constraints to PA (e.g., too tired to do more PA)	-12.1 (4.5)	**1.60 (0.11, 3.09)**	-11.9 (4.4)	1.28 (-0.07, 2.63)	-12.2 (4.4)	-0.48 (-1.21, 0.25)	-11.8 (4.5)	-0.33 (-1.12, 0.46)
**Social level**								
*Parental influence*								
Parental concerns about child’s PA/SB	-3.9 (3.3)	**4.59 (2.51, 6.66)**	-4.4 (3.1)	**7.47 (4.90, 10.04)**	-3.8 (3.3)	0.57 (-0.44, 1.57)	-4.3 (3.0)	0.59 (-0.69, 1.87)
Parental constraints to child’s PA	6.9 (3.1)	**2.87 (0.36, 5.38)**	6.7 (3.2)	**5.05 (3.19, 6.92)**	6.9 (3.0)	0.14 (-1.01, 1.28)	6.8 (3.1)	0.94 (-0.19, 2.07)
Parent likes to participate in outdoor play	73.5%	4.70 (-8.72, 18.12)	70.8%	7.03 (-7.35, 21.41)	73.0%	3.65 (-3.37, 10.67)	70.0%	-5.31 (-13.37, 2.75)
Parent prefers to be social with other parents	36.7%	-5.65 (-17.2, 5.90)	39.1%	11.65 (-1.77, 25.07)	35.6%	-0.68 (-7.52, 6.15)	36.8%	-2.02 (-10.21, 6.16)
Parent gets bored watching child playing in outdoor spaces	11.8%	**-22.01 (-36.87, -7.15)**	12.5%	5.81 (-18.58, 30.19)	11.5%	-0.71 (-8.44, 7.02)	12.7%	-7.79 (-18.44, 2.86)
Parent likes child to do activities of older children	24.0%	-10.26 (-24.05, 3.52)	22.5%	12.73 (-2.27, 27.73)	24.7%	1.18 (-6.7, 9.07)	22.0%	6.29 (-2.93, 15.50)
Parent likes child to do activities they did as a child	34.3%	5.27 (-9.72, 20.26)	33.0%	1.79 (-12.19, 15.76)	34.4%	-0.71 (-7.41, 5.99)	34.1%	-0.01 (-8.59, 8.58)
Parent gets bored going to the same place	12.8%	5.71 (-21.47, 32.88)	10.9%	7.73 (-17.83, 33.29)	10.7%	-0.83 (-10.72, 9.06)	11.2%	-6.60 (-16.29, 3.09)
Parent believes it’s important to be active as a family	90.8%	**-26.67 (-48.93, -4.42)**	86.9%	-14.56 (-34.31, 5.19)	90.6%	-0.22 (-12.23, 11.80)	86.8%	-6.87 (-19.39, 5.65)
Parental self-efficacy to support PA	5.9 (1.7)	**-3.62 (-7.02, -0.23)**	5.9 (1.8)	**-8.07 (-11.75, -4.38)**	5.9 (1.7)	0.06 (-2.00, 2.12)	5.8 (1.8)	-0.54 (-2.60, 1.53)
Parental self-efficacy to limit screen time	8.7 (2.3)	**-9.15 (-11.68, -6.62)**	8.9 (2.5)	**-8.25 (-10.98, -5.52)**	8.8 (2.3)	-0.20 (-1.86, 1.46)	9.0 (2.4)	-0.89 (-2.43, 0.66)
Parental health knowledge/beliefs of child's PA	1.5 (2.2)	-4.19 (-8.74, 0.36)	1.3 (2.2)	**-5.65 (-8.87, -2.44)**	1.6 (2.1)	-0.08 (-1.72, 1.56)	1.3 (2.2)	0.46 (-1.31, 2.23)
*Rules and boundaries*								
Parental rules to limit screen time	2.1 (1.6)	**-11.40 (-16.32, -6.48)**	2.2 (1.5)	**-14.20 (-18.24, -10.16)**	2.2 (1.6)	-0.64 (-2.65, 1.37)	2.2 (1.5)	-1.90 (-4.46, 0.66)
Parental rules about games inside (e.g., no throwing balls inside)	-0.2 (2.1)	1.13 (-2.50, 4.76)	-0.2 (2.1)	1.13 (-2.50, 4.76)	-0.2 (2.1)	-0.16 (-1.90, 1.58)	-0.1 (2.1)	1.16 (-0.73, 3.05)
Parental rules about PA for stranger danger, traffic, injury	2.3 (1.5)	-1.23 (-6.15, 3.70)	2.3 (1.6)	1.27 (-2.65, 5.19)	2.3 (1.5)	-1.73 (-4.17, 0.71)	2.3 (1.5)	-0.31 (-3.17, 2.54)
Parent allows child to play freely in backyard/street	-0.1 (1.2)	-6.29 (-13.74, 1.16)	-0.1 (1.2)	**-7.11 (-12.49, -1.73)**	-0.1 (1.1)	-2.67 (-6.09, 0.75)	-0.04 (1.2)	-0.97 (-3.99, 2.05)
Parent switches off screen entertainment	2.7 (1.4)	3.19 (-0.62, 7.00)	2.6 (1.3)	3.26 (-1.76, 8.28)	2.7 (1.4)	-0.27 (-2.80, 2.26)	2.7 (1.3)	-0.83 (-3.35, 1.69)
*Social interaction and support*								
Child is active at social gatherings	6.0 (1.0)	5.26 (-1.42, 11.95)	6.0 (1.2)	1.05 (-5.88, 7.97)	6.0 (1.0)	-0.54 (-5.45, 4.37)	5.9 (1.2)	-1.86 (-4.50, 0.77)
Maternal PA emotional support for child	5.5 (2.1)	**4.32 (1.74, 6.90)**	5.4 (2.1)	1.20 (-1.84, 4.24)	5.5 (2.1)	**-1.75 (-3.46, -0.04)**	5.4 (2.1)	-0.92 (-2.55, 0.71)
Paternal PA emotional support for child	4.7 (2.4)	**4.29 (1.89, 6.70)**	4.6 (2.4)	1.86 (-0.95, 4.66)	4.7 (2.4)	-1.25 (-2.77, 0.28)	4.6 (2.4)	-0.73 (-2.43, 0.97)
*Modelling of PA*								
Maternal time in PA (hours/week)	5.4 (4.2)	-0.01 (-1.72, 1.69)	5.0 (4.0)	-1.28 (-3.01, 0.45)	5.3 (4.0)	-0.17 (-0.79, 0.45)	4.9 (3.9)	0.46 (-0.54, 1.45)
Paternal time in PA (hours/week)	5.4 (4.7)	0.24 (-0.93, 1.40)	5.2 (4.6)	**-2.12 (-3.65, -0.59)**	5.0 (4.3)	-0.47 (-1.17, 0.22)	5.2 (4.5)	0.35 (-0.53, 1.23)
Maternal TV viewing (hours/week)	8.6 (6.8)	**3.87 (1.81, 5.93)**	9.0 (6.5)	**2.62 (1.56, 3.68)**	8.6 (6.1)	-0.14 (-0.74, 0.46)	9.2 (6.6)	0.10 (-0.46, 0.66)
Paternal TV viewing (hours/week)	9.6 (6.6)	**1.91 (0.96, 2.85)**	9.8 (7.0)	**2.26 (1.23, 3.29)**	9.8 (6.4)	0.16 (-0.39, 0.71)	9.6 (6.9)	-0.01 (-0.48, 0.46)
Maternal role modelling of PA (times/week)	3.2 (2.0)	-0.63 (-4.49, 3.23)	3.0 (2.0)	**-3.67 (-6.97, -0.38)**	3.3 (2.0)	-0.29 (-1.94, 1.35)	3.0 (2.0)	-1.03 (-3.27, 1.22)
Paternal role modelling of PA (times/week)	2.8 (2.0)	-1.82 (-4.88, 1.24)	2.6 (2.0)	**-4.68 (-7.73, -1.63)**	2.8 (1.9)	-0.21 (-2.01, 1.60)	2.7 (2.0)	-0.28 (-2.26, 1.69)
**Physical environment level**								
Dog ownership	32.3%	3.35 (-9.64, 16.34)	32.2%	-1.44 (-14.89, 12.00)	32.1%	0.90 (-6.43, 8.22)	31.2%	4.94 (-2.91, 12.79)
Number of pieces of toys/equipment to be physically active with at home (e.g., swings, slide)	13.3 (3.5)	-1.62 (-3.25, 0.001)	13.1 (3.7)	**-2.37 (-4.15, -0.60)**	13.4 (3.5)	-0.12 (-1.17, 0.93)	13.1 (3.7)	-0.69 (-1.89, 0.52)
Lives on medium/large block	86.1%	1.16 (-14.99, 17.30)	85.2%	-8.11 (-26.65, 10.43)	86.1%	-1.06 (-12.25, 10.13)	84.5%	-6.63 (-18.48, 5.21)
Number of features at home (e.g., front fence, covered outdoor areas)	1.9 (0.9)	**-11.00 (-20.38, -1.62)**	1.9 (0.9)	**-11.48 -17.82, -5.14)**	2.0 (0.8)	2.02 (-2.10, 6.14)	1.9 (0.8)	-3.14 (-8.23, 1.95)
Lives on a cul-de-sac	24.1%	**24.23 (7.27, 41.19)**	29.6%	13.57 (-1.66, 28.79)	23.3%	-5.76 (-13.49, 1.97)	28.0%	0.31 (-7.40, 8.01)
Number of pieces of electronic equipment at home (e.g., DVD player, PlayStation)	5.1 (1.2)	3.83 (-2.76, 10.41)	5.1 (1.1)	2.89 (-2.67, 8.46)	5.1 (1.1)	1.01 (-2.89, 4.91)	5.0 (1.1)	1.58 (-2.33, 5.49)
Number of TVs at home	2.2 (1.2)	**7.31 (1.89, 12.74)**	2.2 (1.1)	**14.08 (7.61, 20.55)**	2.2 (1.1)	-0.80 (-3.60, 2.00)	2.2 (1.0)	**4.10 (1.00, 7.20)**
TV in child’s bedroom	10.6%	**23.51 (4.75, 42.28)**	9.5%	**50.01 (23.84, 76.18)**	10.0%	0.02 (-11.97, 12.01)	7.1%	12.36 (-1.91, 26.63)
Computer/e-games in child’s bedroom	4.2%	11.34 (-28.46, 51.15)	4.2%	5.86 (-22.70, 34.41)	4.1%	17.37 (-9.11, 43.85)	3.7%	15.80 (-1.21, 32.81)
Neighbourhood playground suitability (e.g., equipment, shade, safety)	5.5 (4.4)	**-2.98 (-5.30, -0.66)**	5.2 (4.4)	**-2.71 (-4.15, -1.28)**	5.6 (4.2)	**-0.92 (-1.65, -0.19)**	5.5 (4.5)	-0.31 (-1.13, 0.52)
Neighbourhood constraints to active transport (e.g., busy roads)	4.9 (4.4)	-1.46 (-3.19, 0.28)	4.8 (4.6)	**-2.06 (-3.50, -0.62)**	5.0 (4.4)	**-0.65 (-1.28, -0.01)**	5.0 (4.4)	-0.42 (-1.34, 0.50)
Total frequency of visiting active places per week	6.5 (3.2)	0.64 (-1.58, 2.85)	5.9 (3.0)	0.24 (-2.62, 3.10)	6.4 (3.2)	-0.77 (-1.90, 0.37)	5.9 (3.1)	-0.35 (-1.60, 0.90)

*BMI* body mass index; *CI* confidence interval; *e-games* electronic games; *PA* physical activity; *SB* sedentary behaviour; *TV* television

Notes: ^a^All models adjusted for age, preschool/childcare attendance and clustering by centre of recruitment; ^b^Reported as % for binary/categorical variables and mean (SD) for continuous variables; ^c^Directly measured height and weight, calculated using Cole et al. classifications; ^d^Parents’ self-reported height and weight, calculated using WHO classifications; ^e^Includes part- and full-time paid employment; ^f^ In Australia, playgroups are informal gatherings for parents (and caregivers) and their children prior to the commencement of school; bolded data indicates significance (*p*<0.05); **-** indicates variable not included in combined model

**Table 2:** Combined regression models^a^ for correlates of screen time and sedentary time for boys and girls

Variable	Screen time mins/day		Sedentary time mins/day	
	Boys (*n*=504)	Girls (*n*=433)	Boys (*n*=394)	Girls (*n*=323)
	β (95% CI)	β (95% CI)	β (95% CI)	β (95% CI)
**Individual level**				
*Demographic and family profile*				
Child disability/poor health	-	-	-	10.18 (-3.03, 23.40)
Child sleep duration (hours)	**-7.49 (-13.46, -1.52)**	-5.67 (-11.57, 0.23)	-3.97 (-7.95, 0.01)	**-5.76 (-8.83, -2.69)**
Mother born in Australia	0.99 (-11.45, 13.42)	**-15.66 (-28.97, -2.35)**	-	-
Maternal BMI category^c^				
Healthy weight (ref)	-	0	-	0
Overweight	-	1.56 (-12.22, 15.34)	-	8.55 (-0.90, 18.00)
Obese	-	15.11 (-4.11, 34.32)	-	5.20 (-4.05, 14.45)
Maternal disability/poor health	-	-	-	10.97 (-5.36, 27.30)
Maternal education				
Year 10 or equivalent (ref)	-	0	-	-
Year 12/trade/diploma	-	-3.39 (-27.86, 21.09)	-	-
University degree/post-graduate	-	-2.62 (-30.91, 25.66)	-	-
Low income status (health care/pension card)	-	-4.22 (-23.18, 14.74)	-	-
Paternal age (years)	-	-	**0.73 (0.10, 1.35)**	-
Father born in Australia	-	-3.93 (-17.82, 9.95)	-	-
Paternal BMI category^c^				
Healthy weight (ref)	0	-	-	-
Overweight	-1.15 (-13.03, 10.74)	-	-	-
Obese	2.79 (-12.80, 18.37)	-	-	-
Paternal education				
Year 10 or equivalent (ref)	-	0	-	-
Year 12/trade/diploma	-	**23.26 (0.11, 46.41)**	-	-
University degree/post-graduate	-	9.66 (-16.33, 35.65)	-	-
*Child PA and SB*				
Usual frequency of active transport per week (e.g., ride a bike to kinder)	-	**-1.72 (-3.14, -0.30)**	-	-
Number of organised activities per week (e.g., swimming, tennis)	-	-7.54 (-15.72, 0.64)	-	-
*Child personality, preferences and constraints*				
Child is active for longer with someone else	-10.63 (-23.18, 1.93)	-	-	-
Child preferences for SB (e.g., more likely to watch TV than be active)	3.52 (-1.49, 8.53)	**7.09 (2.47, 11.70)**	-	-
Child constraints to PA (e.g., too tired to do more PA)	-0.69 (-2.12, 0.75)	-	-	-
**Social level**				
*Parental influence*				
Parental concerns about child’s PA/SB	1.87 (-0.05, 3.78)	**3.19 (0.62, 5.76)**	-	-
Parental constraints to child’s PA	0.74 (-1.36, 2.84)	1.29 (-0.55, 3.13)	-	-
Parent gets bored watching child playing in outdoor spaces	**-14.43 (-27.99, -0.86)**	-	-	-
Parent believes it’s important to be active as a family	6.08 (-10.86, 23.02)	-	-	-
Parental self-efficacy to support PA	0.76 (-2.78, 4.30)	1.67 (-2.23, 5.58)	-	-
Parental self-efficacy to limit screen time	**-6.52 (-9.51, -3.54)**	**-2.64 (-5.12, -0.16)**	-	-
Parental health knowledge/beliefs of child's PA	-	0.22 (-3.67, 4.11)	-	-
*Rules and boundaries*				
Parental rules to limit screen time	**-5.15 (-9.20, -1.11)**	**-5.20 (-9.94, -0.47)**	-	-
Parent allows child to play freely in backyard/street	-	-4.62 (-9.39, 0.16)	-	-
*Social interaction and support*				
Maternal PA emotional support for child	2.14 (-1.47, 5.76)	-	-1.10 (-2.84, 0.63)	-
Paternal PA emotional support for child	2.14 (-0.87, 5.14)	-	-	-
*Modelling of PA*				
Paternal time in PA (hours/week)	-	-0.62 (-2.29, 1.04)	-	-
Maternal TV viewing (hours/week)	**2.27 (1.09, 3.46)**	1.33 (-0.05, 2.70)	-	-
Paternal TV viewing (hours/week)	0.49 (-0.71, 1.69)	0.65 (-0.33, 1.63)	-	-
Maternal role modelling of PA (times/week)	-	0.36 (-3.27, 4.00)	-	-
Paternal role modelling of PA (times/week)	-	0.54 (-3.54, 4.61)	-	-
**Physical environment level**				
Number of pieces of toys/ equipment to be physically active with at home (e.g., swings, slide)	-	0.57 (-1.15, 2.29)	-	-
Number of features at home (e.g., front fence, covered outdoor areas)	-2.51 (-9.95, 4.92)	-2.34 (-9.40, 4.72)	-	-
Lives on a cul-de-sac	11.70 (-1.26, 24.66)	-	-	-
Number of TVs at home	3.65 (-2.04, 9.34)	5.15 (-1.69, 11.99)	-	2.45 (-0.79, 5.70)
TV in child’s bedroom	-1.74 (-23.72, 20.23)	27.14 (-5.49, 59.77)	-	-
Neighbourhood playground suitability (e.g., equipment, shade, safety)	0.11 (-1.10, 1.32)	-0.69 (-2.11, 0.73)	-0.69 (-1.51, 0.12)	-
Neighbourhood constraints to active transport (e.g., busy roads)	-	0.14 (-1.29, 1.57)	-0.26 (-0.96, 0.43)	-

*BMI* body mass index; *CI* confidence interval; *PA* physical activity; *SB* sedentary behaviour; *TV* television

Notes: ^a^All models adjusted for age, preschool/childcare attendance and clustering by centre of recruitment; ^b^Reported as % for binary/categorical variables and mean (SD) for continuous variables; ^c^Parents’ self-reported height and weight, calculated using WHO classifications; bolded data indicates significance (*p<*0.05); **-** indicates variable not included in combined model
