# Supplementary material for: Do the correlates of screen time and sedentary time differ in preschool children?
Source: BMC Public Health. 2017 Mar 29;17:285. doi: 10.1186/s12889-017-4195-x (PMC5372288; doi:10.1186/s12889-017-4195-x)
Supplement: Additional file 1: — Table S1. Potential correlates of sedentary time and screen time included in individual models. (DOCX 22 kb) [file 12889_2017_4195_MOESM1_ESM.docx]

Additional file 1: Table S1 Potential correlates of sedentary time and screen time included in individual models

| Variable^a^ | Used in analyses |
| --- | --- |
| **Individual domain** |  |
| ***Demographic and family profile*** |  |
| Child disability/poor health | Binary: yes, no |
| Child’s birth parents live together | Recoded as dichotomous: other situation (ref), parents live together |
| Child sleep duration (hours) | Continuous: sum of usual night time sleep and day time naps |
| Child has siblings | Recoded as dichotomous: child does not have siblings (ref), child has siblings |
| Child BMI category^b^ | Dichotomous: underweight/healthy weight, overweight/obese |
| Maternal age (years) | Continuous |
| Mother born in Australia | Recoded as dichotomous: other country (ref), Australia |
| Maternal BMI category^c^ | Categorical: normal weight, overweight, obese |
| Mother in paid employment | Recoded as dichotomous: not employed (ref), employed full/part time |
| Maternal disability/poor health | Binary: no (ref), yes |
| Maternal education | Recoded as categorical: year 10 or equivalent (ref), year 12/ trade/apprenticeship/diploma, university degree or higher |
| Low income status (health care/pension card) | Binary: no (ref), yes |
| Paternal age (years) | Continuous |
| Father born in Australia | Recoded as dichotomous: other country (ref), Australia |
| Paternal BMI category^c^ | Categorical: normal weight, overweight, obese |
| Father in paid employment | Recoded as dichotomous: not employed (ref), employed full/part time |
| Paternal disability/poor health | Binary: no (ref), yes |
| Paternal education | Recoded as categorical: year 10 or equivalent (ref), year 12/ trade/apprenticeship/diploma, university degree or higher |
| ***Child PA and SB*** |  |
| Usual frequency of active transport per week(e.g., ride a bike to kinder) | Continuous: times per week; summed score of 6 active transport items |
| Usual frequency of non-organised activities per week (e.g., play in the backyard) | Continuous: times per week; summed score of 7 non-organised activity items |
| Number of organised activities per week (e.g., swimming, tennis) | Continuous: times per week; summed score of 6 organised activity items |
| Playgroup attendance | Binary: no (ref), yes |
| Average outdoor play time hours/day (week and weekend day) | Continuous |
| ***Child personality, preferences and constraints*** |  |
| Child active co-participation preferences (e.g., child is active by him/herself, child is active with his/her friends) | Continuous: summed score of 4 items |
| Child is active for longer with someone else | Recoded as dichotomous: disagree (ref), agree |
| Child is competitive with other children when being active | Recoded as dichotomous: disagree (ref), agree |
| Child prosocial physical activity behaviour (e.g., asks for opportunities to be active) | Continuous: summed score of 5 items |
| Child preferences for sedentary behaviour (e.g., more likely to watch TV than be active) | Continuous: summed score of 3 items |
| Child constraints to physical activity (e.g., too tired to do more physical activity) | Categorical: summed score of 10 items |
| **Social level** |  |
| ***Parental influence*** |  |
| Parental concerns about PA/SB | Continuous: summed score of 4 items |
| Parental constraints to child’s PA | Continuous: summed score of 6 items |
| Prefer indoor to outdoor play spaces | Recoded as dichotomous: disagree (ref), agree |
| Parent likes to participate in outdoor play | Recoded as dichotomous: disagree (ref), agree |
| Parent prefers to be social with other parents | Recoded as dichotomous: disagree (ref), agree |
| Parent gets bored watching | Recoded as dichotomous: disagree (ref), agree |
| Parent likes child to do activities of older children | Recoded as dichotomous: disagree (ref), agree |
| Parent likes child to do activities they did as a child | Recoded as dichotomous: disagree (ref), agree |
| Parent gets bored going to the same place | Recoded as dichotomous: disagree (ref), agree |
| Parent believes it’s important to be active as a family | Recoded as dichotomous: disagree (ref), agree |
| Parental self-efficacy to support PA | Continuous: summed score of 2 items |
| Parental self-efficacy to limit SB | Continuous: summed score of 3 items |
| Parental health knowledge/beliefs of child's physical activity | Continuous: summed score of 3 items |
| ***Rules and boundaries*** |  |
| Parental rules to limit screen time | Continuous: summed score of 2 items |
| Parental rules about games inside (e.g., no throwing balls inside) | Continuous: summed score of 2 items |
| Parental rules about PA for stranger danger, traffic, injury | Continuous: summed score of 2 items |
| Parent allows child to play freely in backyard/street | Continuous: summed score of 2 items |
| Parent takes child outside to play if inside too long | Recoded as dichotomous: disagree (ref), agree |
| Parent switches off screen entertainment | Continuous: summed score of 2 items |
| *Social interaction and support* |  |
| Child is active at social gatherings | Continuous: summed score of 3 items |
| Maternal PA emotional support child | Continuous (times/week) |
| Paternal PA emotional support child | Continuous (times/week) |
| ***Modelling of PA*** |  |
| Maternal time in PA per week (hours/week) | Continuous |
| Paternal time in PA per week (hours/week) | Continuous |
| Maternal TV viewing (hours/week) | Continuous |
| Paternal TV viewing (hours/week) | Continuous |
| Maternal role modelling for child PA | Continuous (times/week) |
| Paternal role modelling for child PA | Continuous (times/week) |
| **Physical environment level** |  |
| Dog ownership | Binary: no (ref), yes |
| Number of pieces of toys/equipment to be physically active with at home (e.g., swings, slide) | Continuous |
| Lives on medium/large block | Recoded as dichotomous: small/none (ref), medium/large |
| Number of features at home (e.g., front fence, covered outdoor areas) | Continuous |
| Lives on a cul-de-sac | Binary: no (ref), yes |
| Number of pieces of electronic equipment at home (e.g., DVD player, PlayStation) | Continuous |
| Number of TVs at home | Continuous |
| TV in child’s bedroom | Binary: no (ref), yes |
| Computer/e-games in child’s bedroom | Binary: no (ref), yes |
| Neighbourhood playground suitability (e.g., equipment, shade, safety) | Continuous: summed score of 6 items |
| Neighbourhood constraints to active transport (e.g., busy roads) | Continuous: summed score of 7 items |
| Total frequency of visiting active places per week | Continuous: summed score of 10 items |

Notes: ^a^ Unless otherwise stated, all measures are assessed by parental proxy-report survey; ^b^ Directly measured height and weight, calculated using Cole et al. classifications; ^c^ Parents’ self-reported height and weight, calculated using WHO classifications

Abbreviations: BMI = body mass index; e-games = electronic games; PA = physical activity; SB = sedentary behaviour; TV = television
